# The Breakthrough of Large Language Models Release for Medical Applications: 1-Year Timeline and Perspectives

**DOI:** 10.1007/s10916-024-02045-3

**Published:** 2024-02-17

**Authors:** Marco Cascella, Federico Semeraro, Jonathan Montomoli, Valentina Bellini, Ornella Piazza, Elena Bignami

**Affiliations:** 1 Anesthesia and Pain Medicine, Department of Medicine, Surgery and Dentistry “Scuola Medica Salernitana”, University of Salerno, Via S. Allende, Baronissi, 84081 Italy; 2 Department of Anesthesia, Intensive Care and Prehospital Emergency, Maggiore Hospital Carlo Alberto Pizzardi, Bologna, Italy; 3 Department of Anesthesia and Intensive Care, Infermi Hospital, AUSL Romagna, Viale Settembrini 2, Rimini, 47923 Italy; 4 Anesthesiology, Critical Care and Pain Medicine Division, Department of Medicine and Surgery, University of Parma, Viale Gramsci 14, Parma, 43126 Italy

**Keywords:** Large Language Models, Chatbot, Natural Language Processing, Artificial Intelligence, ChatGPT, Clinical Decision Support, Generative AI

## Abstract

Within the domain of Natural Language Processing (NLP), Large Language Models (LLMs) represent sophisticated models engineered to comprehend, generate, and manipulate text resembling human language on an extensive scale. They are transformer-based deep learning architectures, obtained through the scaling of model size, pretraining of corpora, and computational resources. The potential healthcare applications of these models primarily involve chatbots and interaction systems for clinical documentation management, and medical literature summarization (Biomedical NLP). The challenge in this field lies in the research for applications in diagnostic and clinical decision support, as well as patient triage. Therefore, LLMs can be used for multiple tasks within patient care, research, and education. Throughout 2023, there has been an escalation in the release of LLMs, some of which are applicable in the healthcare domain. This remarkable output is largely the effect of the customization of pre-trained models for applications like chatbots, virtual assistants, or any system requiring human-like conversational engagement. As healthcare professionals, we recognize the imperative to stay at the forefront of knowledge. However, keeping abreast of the rapid evolution of this technology is practically unattainable, and, above all, understanding its potential applications and limitations remains a subject of ongoing debate. Consequently, this article aims to provide a succinct overview of the recently released LLMs, emphasizing their potential use in the field of medicine. Perspectives for a more extensive range of safe and effective applications are also discussed. The upcoming evolutionary leap involves the transition from an AI-powered model primarily designed for answering medical questions to a more versatile and practical tool for healthcare providers such as generalist biomedical AI systems for multimodal-based calibrated decision-making processes. On the other hand, the development of more accurate virtual clinical partners could enhance patient engagement, offering personalized support, and improving chronic disease management.

**Supplementary Information:**

The online version contains supplementary material available at 10.1007/s10916-024-02045-3.

## Introduction

Natural Language Processing (NLP) is a subfield of artificial intelligence (AI) that focuses on the interaction between computers and human language. Notably, NLP models can enable machines to understand, interpret, and generate human-like text or speech. Large Language Models (LLMs) are advanced NLP models within the category of pre-trained language models (PLMs), achieved through the scaling of model size, pretraining corpus, and computational resources [1]. Briefly, LLMs are developed through deep learning methodologies, particularly employing transformer architectures. They are neural network models implementing self-attention mechanisms for enabling the model to consider the entire context rather than being restricted to fixed-size windows, and multi-head attention to capturing contextual relationships in input sequences. In this process, recurrent and convolution layers are not required [2]. Other crucial components of the transformer architecture include encoder and decoder structures to respectively process the input sequence and generate the output sequence. Nevertheless, the architecture of a transformer can vary depending on its specific task and design. Some transformers are designed only with an encoder structure, while others may include both encoder and decoder components. For example, in tasks like language translation, where input and output sequences are involved, both encoder and decoder modules are required. Conversely, for language modeling or text classification, only an encoder may be used. Other key elements of a transformer architecture encompass feedforward neural networks for capturing complex, non-linear relationships in the data, and positional encodings to provide information about the positions of tokens in the sequence [3].

One of the key features of LLMs is their ability to learn contextual information from large datasets, enabling them to grasp complex language structures and nuances. Therefore, LLM applications are effectively employed for text understanding, speech recognition, language generation and translation, chatbots and virtual assistance, sentimental analysis, and other purposes.

The widespread integration of OpenAI’s LLM *ChatGPT* (Chat Generative Pre-Trained Transformer) has stirred considerable excitement since its debut in November 2022 [4]. Following this release, a proliferation of new tools throughout 2023, gave rise to a dynamic landscape of technological progress. The architectures and training methods of these instruments differ, and their functionality is partially understandable in terms of model interpretability of inputs/features/outputs, transparency of model architecture, and training methods. In some cases, the weights, i.e., the parameters that the model learns during the training phase and uses for decisions (output), have been disclosed but this is not consistently achievable.

Setting aside technical details, the user-friendly interface, and the availability of open licenses, particularly for basic frameworks, have been key factors in the quick proliferation of these systems. They hold promise for healthcare applications, particularly in the development of chatbots, interaction systems for clinical documentation management, and the summarization of medical literature (Biomedical NLP). The key challenge in this domain is the exploration of applications for diagnostic and clinical decision support, along with patient triage. As healthcare professionals, we acknowledge the urgent imperative to stay on the cutting edge of knowledge. Nevertheless, staying updated with the evolution of this type of technology is practically impossible, and above all, understanding the potential applications remains a subject of debate.

This article aims to provide a concise overview of the LLM tools released in 2023, emphasizing their potential applications in the field of medicine. While the list may not be exhaustive, the publication aims to offer insight into a phenomenon that is progressively transforming the landscape of medicine, influencing research and clinical practices, as well as healthcare processes.

### Development of LLMs for Chatbots and Enhanced human-like Interaction

The innovative transformer architecture has paved the way for the development of various LLMs, each distinguished by its unique characteristics [3]. Recent advancements in language modeling have led to the emergence of three predominant categories, classified based on the fundamental modules employed in their construction. Firstly, there are encoder-only LLMs exemplified by *BERT* (Bidirectional Encoder Representations from Transformers) and its various iterations. These models excel in capturing contextual information bidirectionally, fostering a comprehensive understanding of language semantics. Secondly, decoder-only language models, as epitomized by the GPT family members, emphasize the generation of coherent and contextually relevant sequences. Leveraging unidirectional attention blocks, these models have demonstrated proficiency in tasks requiring sequential understanding and generation. Lastly, encoder-decoder language models, such as *T5* (Text-to-Text Transfer Transformer) and *BART* (Bidirectional and AutoRegressive Transformers), represent a fusion of both bidirectional and unidirectional attention mechanisms. This hybrid approach allows for versatile applications, ranging from text summarization to language translation, where understanding context and generating coherent responses are both crucial. The application of diverse processes and datasets allows for the provision of a spectrum of tools tailored to meet the evolving demands of natural language understanding and generation [5].

Before the rise of LLMs, traditional deep-learning models grappled with numerous technical challenges, including inadequate sequence and semantic understanding, along with computational complexity. This complexity necessitated a substantial number of parameters, as evident in convolutional neural networks, to achieve satisfactory outcomes. Additionally, issues such as vanishing gradients (e.g., in recurrent neural networks) posed challenges in capturing long-term dependencies, while sequential computation impeded the efficiency of training and inference processes, particularly for extended sequences [3]. The true innovation stemmed from optimizing pre-trained language models to suit the specific demands of chat-oriented tasks, thereby achieving enhanced performance in applications that involve dialog-finetuned versions for conversational interactions. This complex process provides the use of different approaches. Chat-based fine-tuning, for instance, refers to the modality of refining a pre-existing LLM through additional training specifically tailored to conversational or chat-based contexts. In this approach, the model is fine-tuned using datasets that consist of dialogues or interactions, often in the form of message-response pairs. Therefore, the model can learn the nuances of natural language interactions, including the flow of conversation, context handling, and the generation of appropriate responses [6]. It can be able to understand user queries, maintain context across turns, and provide contextually relevant and coherent replies. The pre-trained model can be also provided with additional training data specifically tailored to instructions or guidelines. This fine-tuning method helps generate outputs that align more closely with desired instructions, improving its performance in tasks that require explicit guidance [3]. Furthermore, reinforcement learning from human feedback (RLHF) is a process that requires the involvement of humans in ranking the output efficacy (human feedback) [7]. The RLHF process was fundamental to the success of *ChatGPT*. Remarkably, each of the fine-tuning or RLHF steps can be executed either independently or sequentially. This flexibility is particularly relevant considering that many LLM chat models undergo multiple stages of training, ultimately leading to improved performance and effectiveness in various natural language understanding and generation tasks. Finally, the direct preference optimization (DPO) approach is aimed to directly optimize the model for user preferences or desired outcomes. It is proposed to be an alternative to RLHF. Concerning its functioning, DPO bypasses intermediary steps and directly targets the optimization of user-defined criteria. Therefore, the model can focus on more relevant and satisfying results for users achieving personalized and contextually relevant outputs [8].

### 2023 Timeline of LLMs

*Premise*. The recent release of the models has resulted in the disclosure of clinical applications mostly as preprints, with technical notes frequently inferred from companion blog posts. As a consequence, the findings may not undergo validation or be regarded as definitive. Furthermore, the possible lack of precise details on the methodologies, limitations, and nuances of the models must be necessarily considered.

**Fig. 1:**
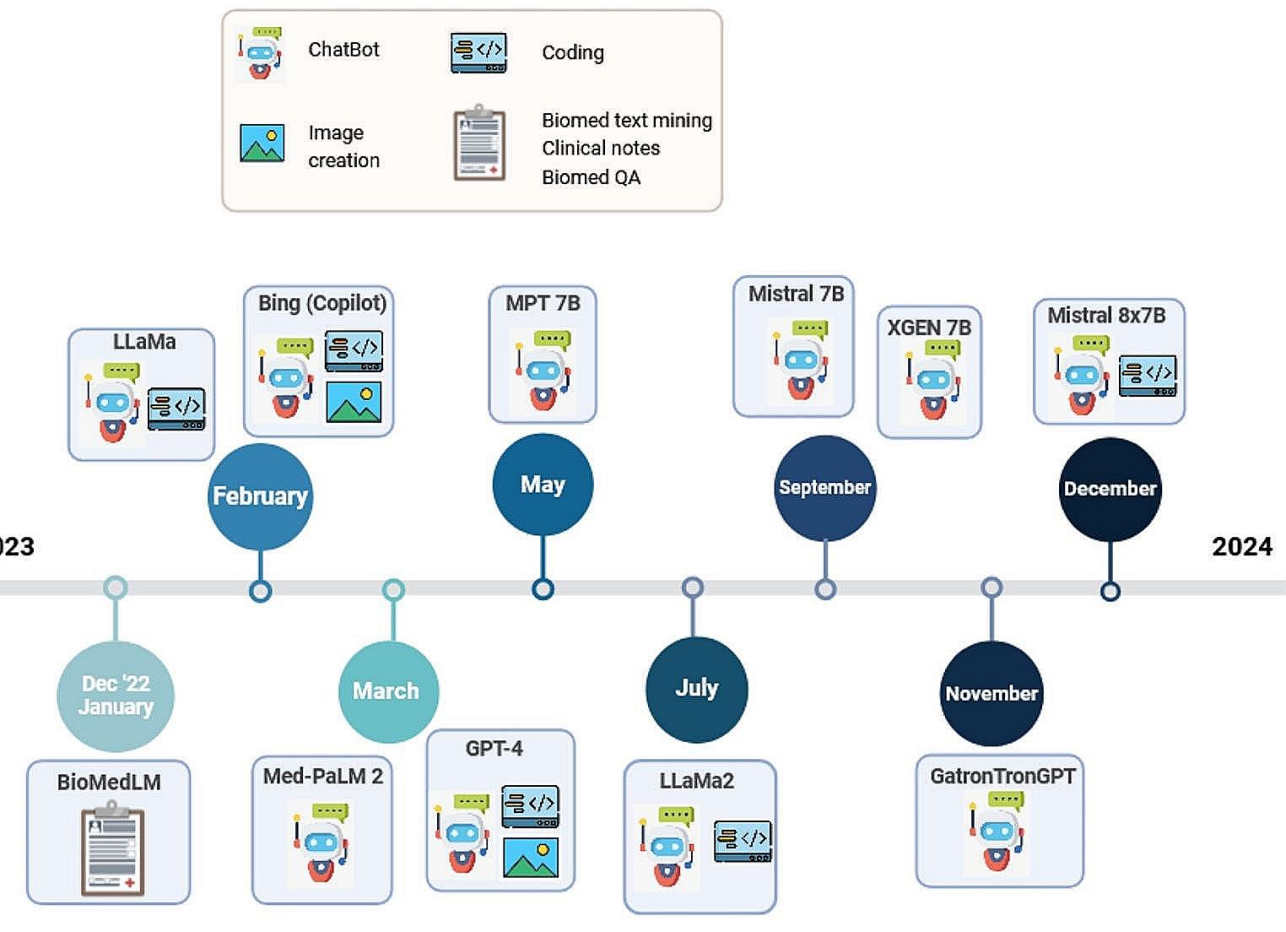
Timeline of selected large language models launched between December 2022 and December 2023

The timeline of the recently released LLMs is illustrated in Fig. 1.

## December 2022-January 2023

At the end of December 2022, a partnership between Stanford CRFM and MosaicML released *BioMedLM* 2.7B (previously indicated as *PubMedGPT* 2.7B). Since it was trained to interpret biomedical language, BioMedLM is a domain-specific LLM for biomedicine. The model was developed by implementing the 825 GiB Pile open-source dataset (16 million PubMed Abstracts and 5 million PubMed Central full-text articles) for language modeling which encompasses 22 smaller, high-quality databases [9]. Trained on the MosaicML Cloud, a platform tailored for handling workloads such as LLMs, the model utilized the Composer training library and PyTorch. Along with GPT, *BioMedLM* 2.7B is an autoregressive language model that generates outputs one token at a time, conditioning each prediction on the previously generated tokens. In other words, the model predicts the next token in a sequence based on the preceding tokens, allowing it to capture dependencies and sequential patterns in the data. Concerning performances, this model can effectively complete various biomedical NLP tasks. For example, it reached a good accuracy on the open domain question answering (OpenQA) dataset examination MedQA [9]. Concerning applications, it is suitable to leverage NLP approaches for understanding and responding to medical-related questions (biomedical Q&A), for clinical notes, and especially for biomedical text mining.

## February 2023

In February 2023, Meta AI introduced *LLaMA* (Large Language Model Meta AI), a suite of fundamental language models spanning a parameter range from 7 billion (7B) to 65 billion (65B). Since models within this range are tailored to cater to a broad array of linguistic intricacies, *LLaMA* products can provide a robust foundation for addressing multiple NLP tasks. Moreover, these models underwent training on trillions of tokens, exclusively utilizing publicly accessible datasets. The weights are provided upon request [10]. In their article, Li et al. [11] illustrated that ChatDoctor, created using an extensive dataset comprising 100,000 patient-doctor dialogues extracted from a widely utilized online medical consultation platform, was able to be proficient in comprehending patient inquiries and offering precise advice. They used the 7B version of the LLaMA model. Nevertheless, despite its limited number of parameters, it demonstrated a performance comparable to the significantly larger GPT-3 model (with 175B parameters).

In this month, Microsoft launched *Bing Chat* (now *Copilot*). The model underwent several updates and shows formidable features such as multimodal input (including images), and code generation. For its working, *Copilot* uses GPT-4 Turbo, and implements Code Interpreter, and DALL-E 3 for coding and image creation, respectively. It can effectively work with the Microsoft suites. For example, the model can generate a draft slideshow with information from another type of file (e.g., word) and synchronize the text with animations already present in a presentation [12]. Regarding performance, ZDNET conducted tests on both *Copilot* and OpenAI’s *ChatGPT* chatbot. They observed that Bing’s version addressed some significant issues usually encountered with *ChatGPT*, such as having knowledge of current events through internet access and providing footnotes with links to sources for the information it retrieved [13]. In the medical context, *Copilot* can be used for different aims. For example, it enables the utilization of an Excel file to monitor the advancement of clinical trials and generate natural language summaries of multimodal clinical information [14].

## March 2023

March 2023 marked an extraordinary milestone in the LLMs era. OpenAI unveiled *GPT-4*, the latest addition to the unidirectional GPT family. This model became accessible to the public through the paid chatbot service ChatGPT Plus and OpenAI’s API. The pre-training phase utilized a combination of public and licensed datasets. The fine-tuning process employed RLHF, reinforcement from artificial intelligence, and ensured policy compliance [15]. Although the data is unconfirmed, it appears that *GPT-4* is built upon eight models, each boasting 220B parameters. Overall, the model is over 10-fold larger than *GPT-3* [16]. The single models are interconnected within the Mixture of Experts (MoE) architecture. This structure represents a form of ensemble learning that amalgamates various models, referred to as “experts,” to arrive at a decision. Within an MoE model, a gating network determines the weighting of each expert’s output based on the input, allowing for specialization in different segments of the input space. This architectural approach proves particularly advantageous for extensive and intricate datasets, effectively partitioning the problem space into more manageable subspaces. The model immediately demonstrated the capability to achieve high performance, showcasing an enhanced conversational experience and improved response accuracy, with fewer hallucination phenomena [17]. It can also manage multimodal data such as images. Therefore, despite not being specifically trained for healthcare or medical purposes, its versatility enables a range of applications in these fields. For example, the model passed a text-based radiology board–style examination [18] and the Korean National licensing examination for clinicians [19]. It was also used for student training [20] and patient education [21]. On the contrary, it was less accurate than trained healthcare personnel in laboratory tasks [22].

Pathways Language Model (*PaLM*) refers to a language model based on the pathway architecture. *Med-PaLM* (Med-Pathways Language Model) is a large-sized AI-powered language model (540B parameter LLM) designed to provide highly accurate answers to medical questions developed by Google, in late 2022. Tailored and tested for the medical domain, it incorporates information from different medical question-answering datasets, research, and consumer queries [23]. *Flan-PaLM* is the instruction-tuned variant of PaLM. The newest iteration, *Med-PaLM2*, was introduced at Google Health’s annual event, in March 2023. It was developed based on *PaLM2*, the language model underlying the Google chatbot, Bard that adopted different models over time, beginning with *LaMDA* followed by *PaLM2*, and finally *Gemini Pro*. For training researchers implemented a collection of text datasets from the internet. This corpus included various sources, such as articles, books, websites, and other textual content. Concerning performance, the model demonstrated a level of expertise comparable to that of a human expert in answering the U.S. Medical Licensing Examination (USMLE)-style questions. Furthermore, as the developers reported, they adopted an innovative ensemble refinement encompassing chain-of-thought prompting and self-refine [24], as a prompting strategy to enhance model reasoning [25]. Expanding on the vision-language model *PaLM-E*, Google has developed (July 2023) a multimodal iteration known as *Med-PaLM M*. This system can synthesize and convey information from images such as chest X-rays, medical images (e.g., dermatology), pathology, and other biomedical data for enabling the diagnostic pathway. MultiMedBench is a comprehensive biomedical benchmark that encompasses various modalities such as medical imaging, clinical text, and genomics. It comprises 14 diverse tasks designed for training and evaluation. As the authors stated, while a robust process of validation in real-world scenarios is needed, this could represent a fundamental step toward the so-called generalist biomedical AI able to interpret and manage multimodal data for decision-making purposes [26].

A multidisciplinary team composed of physicians, hospital administrators, lawyers, and AI researchers has released *Hippocratic AI*. It was developed by implementing an RLHF process using healthcare professionals to train and validate the model [27].

## May 2023

In May 2023, MosaicML launched *MPT-7B* [28]. The model is trained on a large amount of data and can handle structured and unstructured data such as audio and video inputs. The modified transformer architecture incorporates performance-optimized layer implementations and removes context length limits by substituting positional embeddings with Attention to Linear Biases (ALiBi). These adjustments enable the model to be trained with high throughput efficiency and stable convergence. Additionally, MPT models can be efficiently deployed using standard NLP platforms and libraries such as Hugging Face and NVIDIA’s Faster Transformer. The model is licensed for commercial use. Kauf et al. [29] adopted MPT and other LLMs to investigate the dynamics of agent-patient interactions. In addition to the base *MPT-7B*, other three models were released including *MPT-7B-Instruct, MPT-7B-Chat*, and *MPT-7B-StoryWriter-65k+.*

### July 2023

In July 2023, in partnership with Microsoft, Meta introduced a series of models under the name *LLaMa 2*, boasting varying parameter sizes of 7 billion, 13 billion, and an impressive 70 billion. The architecture remains largely unchanged from the original model, albeit with a 40% increase in the amount of data used to train the foundational models. The fine-tuned *LLaMA*, called *LLaMa 2-Chat*, was optimized for dialogue use cases [30]. The architecture closely resembles the initial *LLaMa*, incorporating the addition of Grouped Query Attention (GQA) [31]. The model was trained on a data set encompassing 2 trillion tokens.

## September 2023

A few months later, in September, a French startup with various partnerships, launched *Mistral 7B*, as a European answer to OpenAI. The 7B-parameter model appears to outperform *LLaMa 2* 13B in several benchmarks including reasoning, mathematics, and code generation [32]. These superior performances stem from the sliding window attention (SWA) mechanism. This mechanism empowers the model’s attention system to focus on a sliding window or subset of tokens at a time, rather than attending to the entire sequence of tokens. The result is more efficient processing and improved accuracy, even when utilizing a reduced number of parameters. *Mistral-7B* can be finetuned in a medical chatbot by implementing the NLP working platform Hugging Face and a process of 4-bit quantization with parameter-efficient fine-tuning [33]. On December 2023 Mistral released *Mixtral 8 × 7B*. It is based on the Sparse Mixture of Experts (SMoE) model which is a type of neural network architecture that combines the strengths of both global and local specialization in learning tasks. These architectures offer a flexible and adaptive framework for capturing intricate patterns in data while maintaining computational efficiency [34].

In the same month, September 2023, Salesforce developed *XGen-7B* [35]. This family of LLMs, trained on the in-house JaxFormer library and public domain data such as databricks-dolly-15k, oasst1, and Baize, is better equipped to handle longer document inputs. This capability is achieved through training with the standard dense attention (SDA) transformer for sequences up to a length of 8,000, covering a maximum of 1.5 trillion tokens. In particular, the SDA transformer process helps capture relationships between all tokens in the sequence, providing a more comprehensive understanding of the context. This is particularly useful when dealing with long documents or sequences.

## November – December 2023

During this period, several updates to LLMs, especially for *Copilot*, have been released. In November 2023, researchers from the University of Florida and NVIDIA published a paper for explaining *GatorTronGPT* [36]. The model has a GPT-3 architecture and was trained on the Pile dataset and de-identified clinical text from the University of Florida. It appears to be suitable for creating and assessing healthcare texts, such as clinical notes, medical reports, drug prescriptions, and other medical documents as well as to assess drug-drug interaction, chemical-disease relation, and drug-target links [36].

Selected models and features are shown in Table 1.

**Table 1:** Large Language Models launched between December 2002 and December 2023

Model	Developer	Architecture	Size‡	Availability	Features
BioMedLM [9]	Stanford CRFM and MosaicML	Autoregressive language model. Trained with Flash Attention	2.7B	Licensed under the terms of BigScience Open RAIL-M license°	Training on the Pile 825 GiB dataset. It could not be used for generating text.
LLaMA 2-Chat [10, 11, 30, 31]	Meta AI with Microsoft	Grouped Query Attention transformer	7B-70B	Publicly accessible (1.2 trillion tokens)	Decoder-only Transformer. Massive process of finetuning from human preferences (alignment procedure)
Mistral-7B [32, 33]	Mistral	Transformer leveraging grouped-query attention, and sliding window attention	7B	Open source	Trained on web-extracted tokens. Released under the Apache 2.0 license
Mixtral 8 × 7B [34]	Mistral	Sparse Mixture of Experts	7B	Will be deployed with an open-source deployment stack	Open weights (Apache 2.0 license). strong performance in code generation
MPT-7B [28, 29]	MosaicML	Optimized for fast training and inference (via Flash Attention and Faster Transformer). AliBi	7B	Open source	Open weights (Apache 2.0 license). Works on structured and unstructured data
XGen-7B [35]	Salesforce	Standard dense attention transformer	7B	Apache 2.0 license	Useful with long documents or sequences
Med-PaLM2 [23–25]	Google*	RLHF, compute-optimal scaling, and few-shot learning	540B	N/A	Tailored and tested for the medical domain. Continual learning and iterative refinement
Med-PaLM M [26]	Google	Flexible multimodal sequence-to-sequence encoding	2B, 84B, and 562B	N/A	Multimodal generative model
GPT-4 [15–22]	OpenAI	Mixture of Experts (MoE) (ensemble learning)^	8 × 220B^	Proprietary license	Enhanced conversational experience and response accuracy (less hallucinations)
Copilot§ [12–14]	Microsoft	Uses GPT-4	8 × 220B^	Proprietary license	Text queries for research, writing, assistance, coding, and images.
GatorTronGPT [36]	University of Florida and NVIDIA	GPT-based	5B-20B	N/A	Trained on clinical data. Useful for unlocking patient data from unstructured EHRs.

*Legend*: ‡Number of parameters (the size can also be measured in other quantities such as amount of RAM required for the model); °The license forbids the use of the model “To provide medical advice and medical results interpretation”; ^Features not confirmed [13]; *Google released: BERT (2018), XLNet (2019), GLaM (2021), LaMDA (2022), PaLM (2022), and Minerva (2022); §Released as Bing Chat. *Abbreviations*: ALiBi, Attention with Linear Biases; RLHF, reinforcement learning from human feedback; EHRs: Electronic Health Records.

In addition to the models listed, many other models and software have been produced in the field of NLP. The most interesting aspect concerns the interpretability of the models, focusing on highly controlled setup processes, and training dynamics. For this purpose, for instance, EleutherAI has developed *Pythia*, a suite encompassing 16 LLMs all trained on public data for analyzing LLMs across training and scaling [37].

### Perspectives for a Broader Range of safe and Effective Applications

**Fig. 2:**
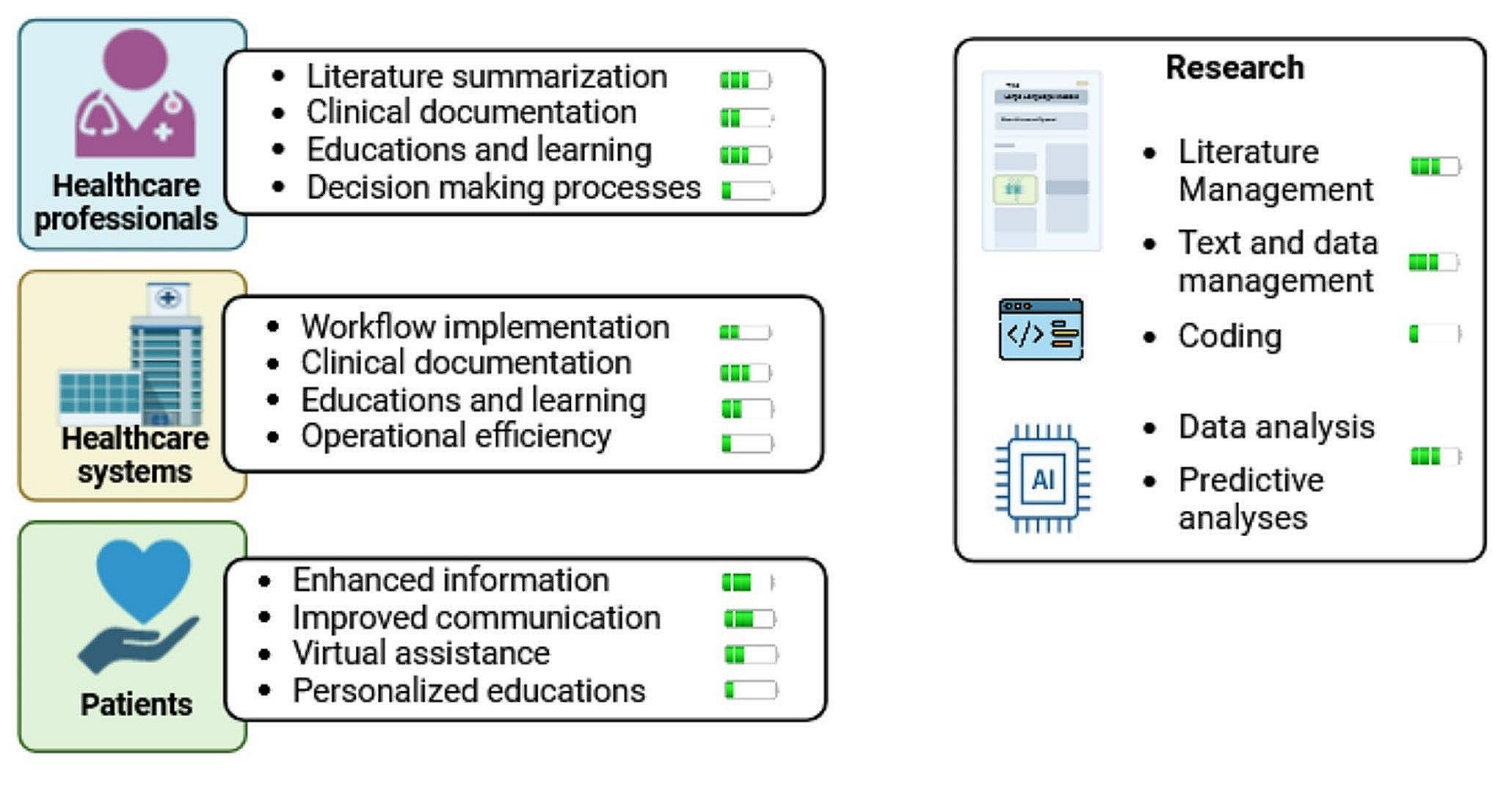
Current applications and perspectives of large language models in medicine. The battery symbol indicates the extent of current applications, ranging from one line to multiple lines

The perspectives in this field encompass broadening the application scope of LLMs and overcoming crucial limitations that hinder the widespread use of these models. Currently, these tools are utilized by clinicians, patients, healthcare systems, and researchers, but it should be noted that the level of development varies across different domains. For instance, tools aiding in writing and literature summarization, such as chatbots, are already widely employed. Conversely, in decision-making processes, we may still be far from their widespread application (Fig. 2).

The healthcare applications of LLMs primarily involve chatbots and interaction systems, clinical documentation, medical literature summarization, as well as diagnostic and clinical decision support. For chatbot use, a limitation arises from the length of the context. Current models struggle to handle increasingly longer contexts while maintaining a high level of reliability and predictability. With the advent of applications like *ChatGPT* and the first version of *Bing Chat* (recently updated and renamed as *Copilot*, Microsoft), users have noticed that the longer they use the model in a single conversation, the more inaccurate its responses become. The cause was the model’s inability to manage context length, leading to confusion and subsequently hallucinations. A series of alternatives are emerging to overcome this limitation. One of these is *Claude*, developed by Anthropic, the AI startup founded by former OpenAI alumni [38]. Moreover, due to the inclusion of GPT-4 Turbo, *Copilot* can manage more than 300 pages of documents in a single prompt. Different research teams from Google developed *Gemini* which is available in three different sizes including *Gemini Ultra*, *Gemini Pro*, and *Gemini Nano*. *Gemini* was developed to be multimodal. Therefore, the model can seamlessly process, understand, manipulate, and integrate various types of information, including text (also PDFs), images, audio, video, and computer code. Text and image outputs can be produced [39]. The intriguing novelty is its ability to fact-check the responses generated by AI, to ensure they are not the product of hallucination.

Concerning medical literature analysis, documents frequently entail substantial dependencies, such as hyperlinks and references, allowing knowledge to extend across multiple articles. To overcome this lack and establish knowledge links between documents, in 2022, researchers from Stanford University developed *LinkBERT*. The training encompassed two domains: the general domain, utilizing Wikipedia articles with hyperlinks, and the biomedical domain, employing PubMed articles with citation links [40].

The incorporation of LLMs in medical education is an important field of research and development [41]. These approaches may offer alternative learning pathways and can be used for designing interactive tools for medical education, enhancing the learning experience [42]. LLMs can also be harnessed for generating case scenarios or quizzes, aiding medical students in practicing and refining their diagnostic and treatment planning skills within a secure and controlled environment [43]. The integration of LLMs in gamification processes represents another captivating perspective [44]. The enhancement of tools for interacting with the patient (virtual clinical partners) could lead to improving patient engagement, providing personalized support, and enhancing chronic disease management [45].

The key perspective revolves around addressing the limitations of LLMs, which encompass challenges such as misinformation, privacy issues, biases in training data, and the risk of misuse [46, 47]. The phenomenon of hallucination can dangerously propagate medical misinformation or introduce biases that have the potential to exacerbate health disparities. In a recent study, for example, Birkun and Gautan [48] showed that the advice provided by LLM chatbots (*Bing*, Microsoft Corporation, USA, and *Bard*, Google LLC, USA) for assisting a non-breathing victim lacked crucial details of resuscitation techniques and, at times, provided misleading and potentially harmful instructions. In another study, carried out to assess the accuracy of *ChatGPT*, Google *Bard*, and Microsoft *Bing* in distinguishing between a medical emergency and a non-emergency, the authors concluded that the examined tools need additional improvement to accurately identify different clinical situations [49]. Continuous verification of the output’s appropriateness is crucial. Significantly, in November 2022, Meta’s *Galactica* model was halted shortly after its release, just a few days after, due to the generation of inaccurate data [50]. The overarching goal is to ensure NLP assurance. This comprehensive process is incorporated at every stage of the NLP development lifecycle, aiming to validate, verify, and make outcomes trustworthy and explainable to non-experts. Additionally, it underscores ethical deployment, unbiased learning, and fairness toward users [51].

In the training phase, the accuracy of the output relies heavily on the choice of the reference dataset [1, 3]. Models like *GPT-4* are generated using extensive data. Nevertheless, data with privacy restrictions, such as those stored in an electronic health record system within a healthcare organization or any medical information exclusive to the private network, are excluded from the training process. Another significant concern pertains to the training and fine-tuning process. The integration of various techniques, such as instruction-tuning and in-prompt strategies like few-shot and chain-of-thought prompting, has notably improved the performance of LLMs. For instance, the authors have introduced an alignment strategy — an approach used to synchronize or align different components of a model or system — specifically tailored for medical question-answering, referred to as ‘expand-guess-refine,’ providing a parameter and data-efficient solution [52].

Taken together, these limitations must be carefully addressed, especially when the output involves complex and paramount tasks such as applications in diagnostic, patient triage, and decision-making processes. For example, Benary et al. [53] showed that different LLMs including *ChatGPT* and *BioMedLM*, are not currently suitable for routine use as tools to assist in personalized clinical decision-making in oncology.

Because of their considerable complexity, these models are often perceived as black-box models. Consequently, a rising concern revolves around the ethical responsibility of deploying such technology [54]. Interpretability aims to convey explanations of the model’s functioning in a manner comprehensible to humans. For this purpose, various approaches have been proposed, categorized as intrinsic models which are constructed with transparency and interpretability considerations as primary design principles, and post-hoc models [55].

As the Med-Palm developers wisely noted, while the results in the field of LLMs in medicine are promising, the medical domain is intricate [24]. Consequently, further assessments are imperative, particularly in terms of safety, equity, and bias. Numerous limitations need to be addressed before LLMs can be considered viable for use in clinical applications [24]. Nevertheless, the correct path appears to be set. To tackle the hallucination phenomenon, for example, Tran et al. [56] implemented a selective prediction task. This involved utilizing the number of decodes matching a given answer from self-consistency as a measure of uncertainty. The researchers applied this measure to withhold the answer if the model lacked sufficient confidence. Other attempts were conducted to align LLMs to the medical domain, working on prompting [57] and prompt tuning [58]. Moreover, efforts are currently underway to devise procedures for evaluating bias and harms associated with fairness [59]. Finally, a multidisciplinary team composed of physicians, hospital administrators, lawyers, and AI researchers is working on *Hippocratic AI*. It will be a safety-focused LLM for healthcare developed by implementing an RLHF process using healthcare professionals to train and validate the model [60].

## Conclusions

In the course of 2023, a significant influx of LLMs has been introduced by diverse developers, underscoring the expansive potential of research in shaping models for the future of healthcare. A crucial aspect of this evolutionary trajectory involves the transformation from an AI-powered model designed solely for answering medical questions to a more extensive practical instrument for healthcare providers. However, realizing this transition mandates substantial additional research efforts by administrators and end-users alike to guarantee the technology’s safety, reliability, efficacy, and privacy. The search for solutions to these obstacles must run parallel to the rapid technological development that will soon lead to the emergence of generalist biomedical AI processes. These strides pave the way for constructing a unified biomedical AI system proficient in interpreting complex, multimodal data to address a myriad of medical and healthcare challenges. In the meantime, Biomedical NLP techniques are primarily employed to aid in manual curation, interpretation, and knowledge discovery within biomedical literature.

### Declarations Section

## Electronic Supplementary Material

Below is the link to the electronic supplementary material.

Supplementary Material 1

## Data Availability

No datasets were generated or analysed during the current study.
