# Supplementary material for: The Breakthrough of Large Language Models Release for Medical Applications: 1-Year Timeline and Perspectives
Source: J Med Syst. 2024 Feb 17;48(1):22. doi: 10.1007/s10916-024-02045-3 (PMC10873461; doi:10.1007/s10916-024-02045-3)
Supplement: Supplementary file 1 — Supplementary Material 1 [file 10916_2024_2045_MOESM1_ESM.doc]

*Dear Professor Jesse Ehrenfeld, MD, MPH*

*Medical College of Wisconsin, USA*

*Editor-in-Chief of Journal of Medical Systems*

Large Language Models are advanced Natural Language Processing models, a subfield of artificial intelligence (AI). One of the key features of LLMs is their ability to learn contextual information from large datasets, enabling them to grasp complex language structures and nuances. Therefore, LLM applications are effectively employed for text understanding, speech recognition, language generation and translation, chatbots and virtual assistance, sentimental analysis, and other purposes.

This article aims to provide a concise overview of the LLM tools released in 2023, emphasizing their potential applications in the field of medicine. The publication aims to offer insight into a phenomenon that is progressively transforming the landscape of medicine, influencing research and clinical practices, as well as healthcare processes.

The manuscript has been read, reviewed and approved by all of the Authors. The authors take responsibility for and agree with the data presented. The Authors have no conflict of interest to declare.

We hope that you find our manuscript of interest and we thank you for the time that you will spend considering our work.

Sincerely,

Valentina Bellini
